# Impact of Physician-Patient Communication in Online Health Communities on Patient Compliance: Cross-Sectional Questionnaire Study

**DOI:** 10.2196/12891

**Published:** 2019-05-13

**Authors:** Xinyi Lu, Runtong Zhang

**Affiliations:** 1 School of Economics and Management Beijing Jiaotong University Beijing China

**Keywords:** patient portals, communication, patient compliance, consumer health information, decision making, physician-patient relations, personal autonomy

## Abstract

**Background:**

In China, the utilization of medical resources is tense, and most hospitals are highly congested because of the large population and uneven distribution of medical resources. Online health communities (OHCs) play an important role in alleviating hospital congestions, thereby improving the utilization of medical resources and relieving medical resource shortages. OHCs have positive effects on physician-patient relationships and health outcomes. Moreover, as one of the main ways for patients to seek health-related information in OHCs, physician-patient communication may affect patient compliance in various ways. In consideration of the inevitable development of OHCs, although they have several shortcomings, identifying how physician-patient communication can impact patient compliance is important to improve patients’ health outcomes through OHCs.

**Objective:**

This study aimed to investigate the impact of physician-patient communication on patient compliance in OHCs through the mediation of the perceived quality of internet health information, decision-making preference, and physician-patient concordance, using an empirical study based on the self-determination theory.

**Methods:**

A research model was established, including 1 independent variable (physician-patient communication), 3 mediators (perceived quality of internet health information, decision-making preference, and physician-patient concordance), 1 dependent variable (patient compliance), and 4 control variables (age, gender, living area, and education level). Furthermore, a Web-based survey involving 423 valid responses was conducted in China to collect data, and structural equation modeling and partial least squares were adopted to analyze data and test the hypotheses.

**Results:**

The questionnaire response rate was 79.2% (487/615) and the validity rate was 86.9% (423/487); reliability and validity are acceptable. The communication between physicians and patients in OHCs positively affects patient compliance through the mediation of the perceived quality of internet health information, decision-making preference, and physician-patient concordance. Moreover, physician-patient communication exhibits similar impacts on the perceived quality of internet health information, decision-making preference, and physician-patient concordance. Patients’ decision-making preference shows the weakest impact on patient compliance compared with the other 2 mediators. Ultimately, all 3 mediators play a partially mediating role between physician-patient communication and patient compliance.

**Conclusions:**

We conclude that physician-patient communication in OHCs exhibits a positive impact on patient compliance; thus, patient compliance can be improved by guiding physician-patient communication in OHCs. Furthermore, our findings suggest that physicians can share high-quality health information with patients, discuss benefits, risks, and costs of treatment options with patients, encourage patients to express their attitudes and participate in health-related decision making, and strengthen the emotional connection with patients in OHCs, thereby decreasing patients’ misunderstanding of information and increasing concordance between physicians and patients. OHCs are required to not only strengthen the management of their published health information quality but also understand users’ actual attitudes toward information quality and then try to reduce the gap between the perceived and actual quality of information.

## Introduction

### Background

As a type of virtual community, online health communities (OHCs) are developed with the Web 2.0 technology [1,2]. OHCs are platforms for people to communicate with one another regarding health-related topics, thereby promoting the interaction between physicians and patients [3]. OHCs can break through time and space limitations as users can communicate through posts and Web-based messages without meeting each other or chatting in real time [2]. From the perspective of users, OHCs can be divided into 3 categories: (1) for patients to discuss illnesses, share experiences, and exchange information, (2) for physicians to exchange their professional knowledge, and (3) for patients and physicians, specifically, patients can communicate with physicians and seek help from physicians, and physicians can answer patients’ questions and publish common health-related knowledge. This paper mainly focuses on the third category and studies the physician-patient communication in OHCs.

In OHCs, patients can conveniently ask for physicians’ help anytime and anywhere in 2 main ways: posts and one-to-one communication. Therefore, patients can diagnose some simple symptoms by themselves on the basis of the information obtained from OHCs, and their privacy can be protected as the communication is not face to face [3-5]. In terms of functions, OHCs not only provide health information but also provide social support to patients [6]. For example, some psychological needs, such as self-esteem and self-efficacy, can be satisfied [7]. Patients with chronic diseases, such as diabetes, hypertension, and obesity, especially mental diseases, such as depression and schizophrenia, are more dependent on OHCs because of emotional communication [8-11]. However, OHCs also have several drawbacks. Physicians may be less likely to understand patients’ illnesses because of the lack of face-to-face communication. Moreover, the quality of information obtained from OHCs cannot be guaranteed as patients are unable to ensure the identities of physicians, given the zero gatekeeping and zero-cost publishing of the internet [12]. In that case, some patients may be hesitant to seek information or communicate with physicians in OHCs or be hesitant to trust physicians or adopt health-related information provided by physicians in communication. Nevertheless, OHCs are still so popular that an increasing number of patients would like to use OHCs to seek health-related information, to connect with other patients and physicians, and to ask for support [6], because of OHCs’ advantages, such as saving queueing time, developing health management, enhancing physician-patient relationships, and improving health service quality. In consideration of the advantages and inevitable development of OHCs, problems of OHCs need to be improved to assist offline treatments and help patients maintain a healthy lifestyle [7]. Communication is an important way for physicians to provide patients services and for patients to seek health information in OHCs [10]; therefore, we intend to further explore physician-patient communication in OHCs for the purpose of improving treatment efficiency.

In China, the utilization of medical resources is tense, and most hospitals are highly congested because of the large population, uneven distribution of medical resources, and low treatment efficiency. Moreover, medical resources cannot meet the daily needs of residents in some regions of China. OHCs can help alleviate hospital congestions, improve the utilization of medical resources, and relieve medical resource shortages to a certain extent [10,13,14]. Specifically, patients are able to diagnose some simple symptoms by themselves and so do not need to go to hospitals frequently and, therefore, their occupancy of medical resources can be decreased. In addition, physicians can publish health-related articles, answer patients’ questions, and provide advice in OHCs when they are not busy diagnosing patients. Moreover, patients from some medically underdeveloped areas can also acquire help from physicians through OHCs. Physician-patient communication in OHCs has important effects on physician-patient relationships, patients’ satisfaction, and health service accessibility. Atanasova et al [7] summarized that physicians participating in OHCs and communicating with patients can improve patients’ satisfaction, enhance patients’ confidence in physician-patient relationship, and increase the possibility of using health services. Wu and Lu [15] identified the impact of the service provided by physicians in OHCs on patients’ satisfaction and treatment efficiency. Yang et al mainly explored how OHCs can improve treatment efficiency from the perspective of patients’ satisfaction. Sarah et al [16] proposed that physicians are required to actively communicate with patients in OHCs to improve patients’ satisfaction. Petrič et al [17] found that social process, such as communication in OHCs, can affect the relationship between patients and physicians. In general, physician-patient communication in OHCs can help improve treatment efficiency, as physicians can better serve patients if hospital congestions can be alleviated and medical resources can be redistributed and reused through OHCs. In addition to the physician-patient relationship and patients’ satisfaction, patient compliance, which has not been widely studied, is another perspective to improve treatment efficiency through OHCs. Previous studies show that patient compliance can impact the effect of medical regimens and treatments, and high patient compliance is conducive to accelerating patient recovery and increasing treatment efficiency [18-20]. Consequently, the inefficient occupancy of medical resources can be reduced and OHCs can reuse and redistribute these resources. Therefore, this paper intends to discuss how OHCs can influence patient compliance so that we can provide a new way to improve treatment efficiency through OHCs.

OHCs can influence physician-patient relationships [21], and patient compliance is critical in physician-patient relationships and health care. Medical regimens and treatments can be effective if patients can regularly take medicines following prescriptions and keep a healthy lifestyle according to their physicians’ recommendations [22]. Moreover, patients with high compliance tend to be relatively healthier than noncompliant ones [23,24]. Effective communication between physicians and patients can considerably help establish physician-patient relationships and promote effective information exchange, which is beneficial for patients obtaining health information, making suitable decisions, and ultimately producing positive results, such as improving compliance [25]. Therefore, the perceived quality of health information may be a mediator between physician-patient communication and patient compliance. Zolnierek and Dimatteo [26] corroborated that physician-patient communication can influence patient compliance through multiple mechanisms. For instance, effective communication between physicians and patients can bring benefits for support, collaborative relationships, patient-centered interviews, and consequently improving patient compliance. Roberts et al [27] concluded that effective physician-patient communication can lead to high patient compliance. Furthermore, Bultman and Svarstad [28] explored the effect of physician-patient communication on patient compliance through the mediation of satisfaction with treatments. Molfenter and Brown [29] found that patients’ health beliefs play a vitally mediating role between physician-patient communication and patient compliance. Ultimately, Laugesen et al [30] determined the impact of internet health information on patient compliance and proposed that internet health information can improve the communication between physicians and patients and then improve patient compliance. Therefore, there may be several mediators between physician-patient communication in OHCs and patient compliance. In the context of OHCs, we considered the perceived quality of internet health information as one mediator. The internet provides patients new platforms, such as OHCs, to communicate with physicians and promotes them to participate in making health-related decisions [31]; thus, the decision-making preference may also be a mediator between physician-patient communication in OHCs and patient compliance. In the physician-patient relationship, physician-patient concordance is a critical element. High-quality communication can promote the exchanging and understanding of opinions between physicians and patients; therefore, the opinions of patients and physicians may tend to be identical. Laugesen et al [30] identify the significant impact of physician-patient concordance on patient compliance in the context of the internet. Therefore, we take into account the mediation of physician-patient concordance.

This study aimed to identify the impact of physician-patient communication in OHCs on patient compliance from the perspective of psychology, attempting to guide patient compliance through communication in OHCs. As a complex field, behavioral psychology has received attention from researchers in recent years, especially for its application in the study of health [32]. However, patient compliance, which is a dynamic parameter and may be easy to change because of psychological factors, has not been paid sufficient attention [33,34]. In addition, factors such as basic national conditions and national policies have created a unique medical system in China, which is considerably different from foreign medical systems. Therefore, the theoretical and practical results of OHC services in foreign countries cannot be directly applied to Chinese scenarios. Research on OHCs in China is still in its infancy. In this study, on the basis of the self-determination theory, we intended to explore how physician-patient communication in OHCs affects patient compliance through mediations of the perceived quality of internet health information, decision-making preference, and physician-patient concordance to fill in the gap of research and practice.

### Physician-Patient Communication

Collaborative medical interactions exhibit considerable relevance to health care outcomes [35]; thus, physician-patient communication plays an important role in the health care system. Thomas et al [36] claim that physician-patient communication remarkably affects patient response to treatments and, consequently, the quality of diagnoses. Therefore, high-quality physician-patient communication brings benefits to improving health care quality and patients’ health outcomes [37,38], and the communication quality has been regarded as one of the critical elements of health literacy. With the development of the internet, a new form of physician-patient communication, internet communication, has emerged. Patients are increasingly willing to communicate with physicians on the Web because of the advantages of physician-patient internet communication [39]. For instance, the internet provides patients a platform to seek health-related information to self-manage and self-monitor their health and diagnose some nonurgent medical problems [40,41]. In that case, patients may not have to wait for a long time for a simple illness, and congestions in hospitals are likely to be alleviated [10,13,14].

OHCs are one of the main channels for patients to communicate with physicians on the Web. On the one hand, patients can engage with physicians without going to hospitals instead of only seeking health-related information from the internet. In addition, patients can also obtain additional information about their physicians if they contact the physicians before going to the hospital, which may reduce the uncertainty and sense of risk [14]. On the other hand, the majority holds the view that the internet helps individuals improve their abilities of communication [42]. Moreover, between patients and physicians, communicating without meeting each other is less likely to cause conflicts, thereby strengthening the physician-patient relationship. Accordingly, patients are likely to be satisfied and trust their physicians [14,43] and may be willing to comply. However, physician-patient communication in OHCs exhibits shortcomings, which may negatively influence patient compliance in turn. For instance, accurately diagnosing patients without face-to-face communication is difficult for physicians, as they cannot observe patients’ breath, sound, or facial expressions [40]. In this situation, patients may regard their physicians as unprofessional and then refuse to comply with treatments [44]. Therefore, this paper aimed to explore how to guide physician-patient communication in OHCs to improve patient compliance.

### Patient Compliance

Generally, the effect of treatments depends on 2 aspects: (1) whether the treatment proposed by physicians is the correct remedy and (2) whether patients comply with the treatment [30]. Given that we cannot control the degree of physicians’ professionalism, we pay increasing attention to patient compliance to improve the curative effects. Patient compliance is used to measure how patients follow medical diagnoses and treatment regimens recommended by their physicians [30], including medicine adherence and maintaining a healthy lifestyle [33]. Moreover, patient compliance plays a vital role in physician-patient relationships and health care systems, especially from the perspective of chronic diseases, whose recovery relies on patients’ self-management and self-monitoring [23]. If medical diagnoses and treatment regimens are proper, treatments can be increasingly effective when patients’ daily habits are consistent with physicians’ advice [22]. Therefore, patients with high compliance are likely to be relatively healthier than those with low compliance [23]. Khera et al [45] and Johal et al [46] validated that a healthy lifestyle is good for preventing cardiovascular diseases. With the increasing number of patients with chronic diseases, patient compliance becomes increasingly important.

High compliance is conducive to patients’ health outcomes, whereas low compliance or noncompliance may cause negative consequences related to patients, the economy, and the society. Diseases may be hard to treat if patients refuse to comply with physicians. According to Varleta et al [47], blood pressure may be difficult to control without regularly taking medicines following prescriptions. In that case, medical resources will be wasted, given that they fail to work. Moreover, some types of medicines or therapies may be regarded as invalid, and then their use may be terminated. When this phenomenon occurs in clinical practice, medical productivity may be negatively influenced [48,49]. Furthermore, patient compliance is a dynamic parameter [34]; thus, this study aimed to discuss various methods to improve patient compliance.

### Perceived Quality of Internet Health Information

Benefitting from the development of the internet [50], people can gain access to internet-based health-related information that is divided into 2 categories [51-53]: (1) health care information, which is related to diseases, medicines, physicians, hospitals, and therapies and (2) health lifestyle information, which guides not only patients but also all individuals to keep a healthy lifestyle and prevent diseases. Numerous portals have been built by governments, medical institutions, and business corporations to publish various health information on the Web [54], and OHCs are one of the main types of these portals. For specific needs [55], patients enter OHCs to obtain health information in 2 ways: searching for information and communicating with physicians. Physician-patient communication in OHCs is patient-active. If physicians do not respond to patients, it cannot be considered as an entire physician-patient communicating process. Therefore, in this study, patients can be regarded as having obtained health information from physicians as long as they communicate with physicians, regardless of the quality and quantity of information. Compared with the health information from other sources, internet health information can be published on time and be obtained conveniently and quickly because of zero gatekeeping and zero-cost publishing [56]. However, internet health information lacks monitoring, and the quality of internet health information has become a serious problem [57].

Individuals with different health literacy levels exhibit different levels of ability to distinguish the quality of internet health information and perceive different information quality. From the perspective of information users, the perceived quality is different from the actual quality. Sporadically, a person may encounter a piece of high-quality health information related to a specific topic but may consider this information low in quality, in other words, the perceived quality is low. This situation may result from many factors. For instance, the channel that publishes this information may be low in quality and cannot convince users. In addition, the person’s cognition on this topic may be wrong; thus, he or she may consider the information to be low in quality.

### Decision-Making Preference

The internet provides patients with opportunities to contact physicians and obtain health information. Thus, some patients’ health literacy can be improved, and they may be willing to play active roles in health-related decision making [58]. A survey conducted by Jr et al [59] verifies that participating in decision-making activities helps patients achieve improved health outcomes. In addition, Beaver et al [60] proved that patients with psychological diseases are less likely to relapse if they participate in decision making. Therefore, an increasing number of patients are encouraged to be involved in decision making in treatments [59]. However, in the meantime, some patients prefer to rely on their physicians and want physicians to be the main or only decision maker [61]. From the viewpoint of some patients, some physicians may dislike making shared decisions with patients, and therefore, patients cannot participate in decision making; otherwise, physicians may think that their patients are questioning their professions.

Actually, physicians are willing to discuss benefits, risks, and costs of treatment options with patients, encourage patients to positively participate in decision making, and make decisions after considering patients’ views [61,62], which is beneficial for the improvement of patient satisfaction, enhancement of physician-patient relationship, and improvement in health care quality [62]. Participating in decision making does not indicate that patients make decisions by themselves regardless of physicians’ professional advice. On the contrary, participation requires sufficient communication between physicians and patients [63]. When patients’ opinions are contrary to physicians’ opinions, physicians need to patiently explain to patients and make efforts to be consistent with patients. Moreover, as the core of patient-centered care [64], shared decision making plays a key role in high-quality physician-patient communication [65]. Even if patients do not intend to make decisions by themselves, they still want to know treatment options from their physicians. Hence, physicians are required to positively understand their patients’ preferences of decision making instead of waiting for patients’ inquiries.

Patients’ preference for participating in making decisions is dynamic [66]. Vogel et al [67] confirmed that lung cancer patients’ decision-making preference could be stable in the short term but remarkably change after more than 3 months. A number of researchers carried out studies to identify the factors that influence patients’ decision making. Furthermore, Cajita et al [68] explored that some demographic factors (age, gender, education level, and health condition) can affect a patient’s decision-making preference. For instance, patients’ preference for making health-related decisions will change with age. Moise et al [65] concluded that depressive symptoms have an impact on patients’ decision-making preference and that patients with elevated depressive symptoms prefer clinician-directed decision making. In addition, Harvey et al [69] proved that patients’ preferences for participating in decision making vary by their circumstances. Therefore, they proposed that physicians can encourage patients to participate actively in making health-related decisions by sharing information and helping process information to reduce conflicts and improve patient compliance. Currently, patients seek health information on the Web and take the internet as a new open channel to communicate with physicians; thus, they are more willing to participate in decision making [31]. Therefore, decision making may be a mediator between physician-patient communication and patient compliance.

### Physician-Patient Concordance

Physician-patient concordance indicates that patients and physicians equally discuss treatment options [70] and then reach an agreement in terms of medical diagnoses and treatment regimens [21,71], which is important in physician-patient interactions [72]. According to previous studies, when the opinions of patients and physicians tend to be identical, patients’ satisfaction and health care outcomes will be high [72,73]. Patients may be able to manage themselves well and not need further consultation, thereby reducing health care costs [72]. However, a gap about medical knowledge exists between patients and physicians because of the professionalism of medicine; thus, patients’ expectations of health outcomes may sometimes be different from those of physicians, and it may be difficult for physicians to propose medical treatments that are consistent with patients’ values, goals, and priorities [74]. Accordingly, patients’ health outcomes and physician-patient relationships may be negatively affected. Therefore, physician-patient concordance must be improved.

A considerable number of researchers have conducted surveys on physician-patient concordance, comprising its pattern of manifestation, advantages, shortcomings, and influencing factors. Shin et al [73] proved that increased efforts by physicians to understand demands of patients with cancer for the achievement of concordance are beneficial in improving patient compliance. In terms of influencing factors, the impact of patients’ sociodemographic characteristics on concordance has been identified, but other factors have not been fully understood. Moreover, Gross et al [72] deduced that physicians maintaining a long-term relationship with patients can help enhance concordance, whereas ignoring patients’ accurate demands may not directly affect concordance in the consultation process but may affect follow-up treatments.

### Self-Determination Theory

The self-determination theory was proposed by Deci and Ryan [75], and it is the only theory of human motivation that determines autonomy as a human need and has been applied to the fields of health care, education, and organization [76]. According to the self-determination theory [77], when people perceive high autonomy support from social events, their intrinsic motivation of activities will be enhanced [78]. By contrast, perceiving being controlled or forced in social events is more likely to decrease an individual’s intrinsic motivation of activities [79]. In the context of health care, Ng et al [76] inferred that patients’ health-related behaviors may be positively motivated by their autonomy orientations. Specifically, supporting autonomy for patients can satisfy 3 basic aspects of psychological needs (ie, autonomy, relatedness, and competence) [75,77,80] and then patients will begin their health-related behaviors [76], such as quitting smoking, weight control, and medicine adherence [79]. By contrast, if the satisfaction is low, then self-determined motivation may be reduced, and people may involuntarily behave negatively, such as be noncompliant [80].

Autonomous self-regulation plays a critical role in health care, and Ng et al [76] concluded that the self-determination theory is viable to studying the motivation for health-related behaviors. This paper attempted to explore how physician-patient communication in OHCs can impact patients’ motivation and compliance to treatments from the mediating perspective of the perceived quality of internet health information, decision-making preference, and physician-patient concordance by employing the self-determination theory.

### Model and Hypotheses

**Figure 1:**
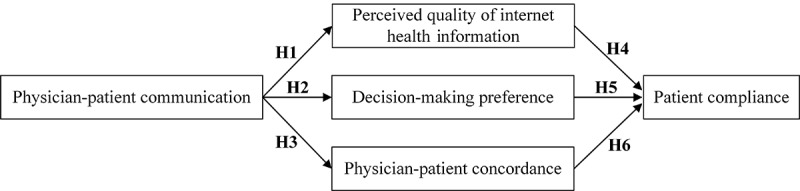
Research model. H1-H6: hypothesis number.

This paper intended to explore the impact of physician-patient communication in OHCs on patient compliance by establishing the research model (see Figure 1), including 1 independent variable (physician-patient communication), 3 mediators (perceived quality of internet health information, decision-making preference, and physician-patient concordance), and 1 dependent variable (patient compliance).

Patient compliance is a dynamic parameter, and noncompliance or low compliance may be involuntary [81]. Therefore, this study focused on patients’ autonomy to improve patient compliance. As one of the main contents of physician-patient communication, information delivery is advantageous to obtaining health-related information and knowledge, helping patients understand their health and conditions and improving patient satisfaction with health care services [10]. Lin et al [43] proved that physician-patient communication on the Web, which relies on an internet-based channel, can be more likely to promote patient satisfaction compared with offline communication [39]. In addition, Web-based communication can help improve patients’ trust in their physicians and the frequency of using OHCs [14]. High trust and satisfaction with physicians make patients perceive autonomy support from physicians, make patients willing to obtain health information from physicians, and make patients likely to trust this information [55]. In that case, internet health information obtained from physicians in OHCs through communication can be fully processed; thus, patients’ psychological demands for the information may be satisfied, and they may perceive high-quality health information. Therefore, we suggested the following hypothesis: (H1) Physician-patient communication has a positive impact on patients’ perceived quality of internet health information.

Promoting physician-patient communication means encouraging patients to inform physicians of their health conditions and urging physicians to discuss treatment options with patients instead of making decisions themselves. A patient’s preference of participating in making decisions is dynamic [66] and thus may change with the progress of communication. The self-determination theory highlights that people who perceive support of autonomy will likely be motivated to change their behaviors [75,79]. When patients and their physicians positively communicate with each other, patients can feel that physicians provide opportunities for them to participate in decision making. Hence, they will perceive autonomy supported by physicians, and then they may actively participate in making decisions and assisting physicians. By contrast, without autonomy support, patients may be overwhelmed by the feeling of being controlled or coerced, which leads to the weakened intrinsic motivation of activities. Accordingly, we proposed the following hypothesis: (H2) Physician-patient communication has a positive impact on patients’ decision-making preference.

Physicians and patients tend to have differences in their cognitions [70], but this divergence may be reduced if they can communicate with each other. Specifically, communication makes physicians understand patients’ health conditions, and patients can feel increased autonomy support from their physicians. In that case, the self-determination theory proposes that patients’ psychological needs can be satisfied and that they are willing to agree with their physicians. In addition, empathy can be produced between physicians and patients, resulting in high concordance. According to Gross et al [72], patients are more likely to be concordant with their long-term physicians, as the communication may be frequent and positive in this relationship. By contrast, if physicians are unwilling to explain benefits, risks, and costs of treatment options or if patients refuse to describe the details of illness, the quality of communication is bad, which may cause suspicion. Moreover, in such an inefficient treatment environment with low support of autonomy, patients may sometimes question their physicians and be reluctant to cooperate with treatments [75]. Consequently, we suggested the following hypothesis: (H3) Physician-patient communication has a positive impact on physician-patient concordance.

Perceiving internet health information as high in quality may make patients think that their physicians are professional and indeed share health-related information with them, thereby enabling patients to feel being allowed or inspired by physicians to participate in medical decision making, and their perceived autonomy support is increased. Under the guidance of the self-determination theory [75], perceived autonomy support may encourage patients to positively change their medical behaviors, such as complying with treatments. In addition, high-quality information may help patients extend the knowledge and experience related to the importance of self-regulation and self-management [76], which can help improve patient compliance with medical diagnoses and treatment regimens. Laugesen et al [30] identified a weak, indirect effect of internet health information quality on patient compliance through mediations of perceived information asymmetry and physician-patient concordance, whereas Lu et al [33] found an indirect effect of internet health information quality on patient compliance by means of the mediating role of affect-based trust. Therefore, the indirect effect of internet health information on compliance may be associated with the mediators, background, sample, and other possible factors. What we can ensure is that the perceived quality of internet health information indeed has an effect on patient compliance. Therefore, we attempted to examine the direct effect of the perceived quality of internet health information on patient compliance. Hence, we proposed the following hypothesis: (H4) Patients’ perceived quality of internet health information has a positive impact on patient compliance.

From the perspective of patients, a high decision-making preference does not mean that they prefer to make decisions by themselves rather than consider physicians’ advice. Generally, physicians mainly decide the final medical options, and patients’ decision-making preference represents the degrees at which patients participate in making decisions. Highly participating in health-related decision making means patients tend to be more autonomous. According to the self-determination theory, autonomy may encourage patients to behave positively in treatments, such as medicine adherence. Therefore, patients who prefer to participate in decision making are likely to perceive autonomy support from their physicians, which may help enhance their internal sense of health care and develop their ability of self-regulation [76], ultimately improving patient compliance with treatments. Therefore, this situation led us to derive the following hypothesis: (H5) Patients’ decision-making preference has a positive impact on patient compliance.

High concordance between physicians and patients is the ideal consequence of treatments. In this situation, patients will highly agree with their physicians; thus, complying with physicians also means following their own choices. Similar to the discussion of the perceived quality of internet health information, patients assume positive attitudes toward their physicians, and they assume a perception of high supportive autonomy that promotes self-regulation of healthy behaviors [76], such as patient compliance, which may be present, according to the self-determination theory. Moreover, Laugesen et al [30] found a direct impact of physician-patient concordance on patient compliance. Hence, we proposed the following hypothesis: (H6) Physician-patient concordance has a positive impact on patient compliance.

## Methods

### Instrument Development

To guarantee reliability and validity, we adopted the previous scales validated by published works to measure variables in the research model (see Figure 1) with a 5-point Likert-type response format that ranged from *strongly disagree* to *strongly agree*, as shown in Multimedia Appendix 1. In the context of OHCs, this study adapted a 14-item scale from Makoul et al [82] to measure the communication between physicians and patients. The perceived quality of internet health information, which was once examined by Laugesen et al [30], was measured using a 16-item scale. In addition, physician-patient concordance and patient compliance under the background of internet health information were also discussed in the study by Laugesen et al [30], with 2 different 5-item scales. To address the subject of this study, we adapted these 2 scales to measure physician-patient concordance and patient compliance in OHCs. Ultimately, the decision-making preference of patients was measured using a 6-item scale by Aoki et al [83].

The next step involved translating the instrument into Chinese, given that the survey would be conducted in China and our subjects were Chinese individuals who have communicated with physicians in OHCs. Referring to the similar translation process by previous studies [84,85], we first recruited native Chinese speakers with at least a master’s degree and who were good at speaking English and scientific research translation to translate the scales into Chinese, considering the cross-cultural adaptation [86]. Second, we invited individuals with experiences in communicating with physicians in OHCs and who were from different backgrounds of ages, genders, and educational levels to complete the questionnaire, provide recommendations for modifying scales, and then improve comprehensibility, conciseness, appropriateness, and readability. The reverse translation process was the last necessary step, which was important to ensure that our scales were conceptually consistent with the original English version [30,87,88].

### Analysis Tool Selection

Structural equation modeling (SEM) is useful in analyzing the causal relationships of research models, including mediators, and accommodating intricate causal networks [30], and it can help incorporate the measurement error and detect effects [89]. This study adopted the partial least squares (PLS)-SEM method to analyze the research model and used SmartPLS version 3.2.8 (SmartPLS GmbH, Bönningstedt, Germany) to analyze the collected data and test hypotheses, drawing lessons from previous studies [30,90].

### Data Collection and Respondent Profile

The subjects of this investigation were Chinese individuals who have communicated with physicians in OHCs within the previous month to ensure that they could recall their relevant experiences. With the help of a medical association in China, the formal investigation was conducted in May 2018, and the questionnaires were sent to 615 participants. The participants’ informed consent was secured, and we committed that their privacy would be strictly protected. We used a Web-based platform to create and maintain the questionnaire, and participants also filled this questionnaire through the platform. This platform can help record the completion time of each response; therefore, the response, whose completion time is obviously lower than the average time, is regarded as invalid. In addition, the response that was not completed or that missed at least 1 answer is also invalid. Finally, we received 487 responses, and 423 of them were valid. Therefore, the response and validity rates were 79.2% (487/615) and 86.9% (423/487), respectively. Table 1 shows the demographics of the sample; we found that 59.3% (251/423) participants were aged 20 to 40 years, 53.6% (220/423) of the participants were female, and 51.3% (217/423) of the participants held at least a bachelor’s degree. Thus, more than half of the subjects were young, female, and highly educated, which was consistent with the characteristics of OHCs’ users and met our requirements [2,91,92].

**Table 1:** Sample demographics (N=423).

Demographic characteristics		n (%)
**Age (years)**		
	<20	19 (4.5)
	20-29	127 (30.0)
	30-39	124 (29.3)
	40-49	97 (22.9)
	50-59	49 (11.6)
	60 and above	7 (1.7)
**Gender**		
	Male	203 (48.0)
	Female	220 (52.0)
**Living area**		
	Urban	240 (56.7)
	Rural	183 (43.3)
**Education**		
	Junior middle school	22 (5.2)
	High school	60 (14.2)
	Junior college	124 (29.3)
	Bachelor’s degree	159 (37.6)
	Master’s degree	48 (11.3)
	Doctorate	10 (2.4)

## Results

### Data Analysis

To identify the effect of demographic factors on relationships in the research model and to adjust the results, we added age, gender, living area, and education level into the research model as control variables. Although this study used previous validated scales to measure variables, we reevaluated the reliability and validity because of differences in backgrounds and participants. We calculated the Cronbach alpha of each construct using SmartPLS software version 3.2.8, as shown in Table 2, and all values were greater than the cut-off value of .700 [88,93], which indicated a good reliability of scales.

Table 3 provides the composite reliability (CR) and the average variance extracted (AVE) of constructs, and Table 4 shows the correlations between each of the 2 constructs. The convergent validity of scales was acceptable, as all the CR values exceeded the cut-off value of .700 and all the AVE values exceeded the cut-off value of .500. For each construct, the square root of AVE was above each correlation between other construct and itself. Therefore, the discriminant validity was acceptable [94,95].

**Table 2:** Cronbach alpha of constructs.

Constructs	Cronbach alpha
Physician-patient communication	.909
Perceived quality of internet health information	.919
Decision-making preference	.749
Physician-patient concordance	.750
Patient compliance	.787

**Table 3:** Composite reliability and average variance extracted.

Construct	Composite reliability	Average variance extracted	Square root of average variance extracted
Physician-patient communication	.940	.527	.726
Perceived quality of internet health information	.947	.528	.727
Decision-making preference	.866	.519	.721
Physician-patient concordance	.869	.570	.755
Patient compliance	.864	.561	.749

**Table 4:** Correlations between each of the 2 constructs.

Construct	PPCOM^a^	PQIHI^b^	DMP^c^	PPCON^d^	PC^e^
PPCOM	1.000	—^f^	—	—	—
PQIHI	.724	1.000	—	—	—
DMP	.624	.665	1.000	—	—
PPCON	.705	.722	.617	1.000	—
PC	.725	.700	.595	.686	1.000

^a^PPCOM: Physician-patient communication.

^b^PQIHI: Perceived quality of internet health information.

^c^DMP: Decision-making preference.

^d^PPCON: Physician-patient concordance.

^e^PC: Patient compliance.

^f^Not applicable.

### Hypothesis Testing

According to the results by SmartPLS 3.2.8, we corroborated that age positively affected patient compliance in OHCs. Specifically, older patients were more willing to comply with medical regimens and treatments recommended by physicians compared with younger patients. In terms of gender, females were more likely to perceive high-quality health information in OHCs and exhibited high compliance with physicians compared with males. Furthermore, the educational level positively affected decision-making preference, indicating that patients who were highly educated would be more likely to participate in health-related decision making. This study used Cohen ƒ^2^ [96] to evaluate the effects of control variables, and Table 5 shows the results of the multivariate coefficient of determination (*R*^2^). Ultimately, we contended that control variables had limited or insignificant effects on the research model.

**Figure 2:**
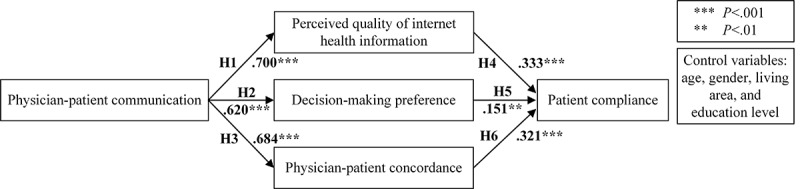
Research model with path coefficients. H1-H6: hypothesis number.

Results of the PLS-SEM can be observed in Figure 2, and Table 6 shows the magnitude and significance of the path coefficients. All 6 hypotheses were supported; specifically, physician-patient communication had positive impacts on perceived quality of internet health information, decision-making preference, and physician-patient concordance. In addition, the perceived quality of internet health information, decision-making preference, and physician-patient concordance all positively affected patient compliance. Table 7 presents the effects of constructs in the research model, which indicates that physician-patient communication had a strong impact, with large effect sizes on the perceived quality of internet health information, decision-making preference, and physician-patient concordance, and the impact of perceived quality of Internet health information, decision-making preference and physician-patient concordance on patient compliance were all weak with small effect sizes.

**Table 5:** Multivariate coefficient of determination (*R*^2^) results.

Variables	*R* ^2^		Control variable effects		
	With control variables	Without control variables	∆ *R*^2a^	ƒ^2b^	Effects
Perceived quality of internet health information	0.532	0.524	0.008	0.017	Insignificant
Decision-making preference	0.394	0.389	0.005	0.008	Insignificant
Physician-patient concordance	0.501	0.496	0.005	0.010	Insignificant
Patient compliance	0.588	0.570	0.018	0.044	Small

^a^*∆R*^*2*^: *R*^2^_with control variables_− *R*^2^_without control variables_.

^b^ƒ^2^: Cohen ƒ^2^.

**Table 6:** Hypothesis testing.

Hypothesis	Path coefficient	*t* test	*P* value
Physician-patient communication has a positive impact on patients’ perceived quality of internet health information	.700	18.693	<.001
Physician-patient communication has a positive impact on patients’ decision-making preference	.620	16.629	<.001
Physician-patient communication has a positive impact on physician-patient concordance	.684	19.677	<.001
Patients’ perceived quality of internet health information has a positive impact on patient compliance	.333	4.569	<.001
Patients’ decision-making preference has a positive impact on patient compliance	.151	3.002	.003
Physician-patient concordance has a positive impact on patient compliance	.321	3.951	<.001

**Table 7:** Partial least squares effect size analysis.

Constructs		*R* ^2a^		∆ *R*^2b^	ƒ^2c^	Effect size
		In	Out			
**Patient compliance**						
	Perceived quality of internet health information	0.588	0.545	0.043	0.104	Small
	Decision-making preference	0.588	0.577	0.011	0.027	Small
	Physician-patient concordance	0.588	0.543	0.045	0.109	Small
**Perceived quality of internet health information**						
	Physician-patient communication	0.532	0.094	0.438	0.936	Large
**Decision-making preference**						
	Physician-patient communication	0.394	0.050	0.344	0.568	Large
**Physician-patient concordance**						
	Physician-patient communication	0.501	0.083	0.418	0.838	Large

^a^*R*^*2*^*:* Multivariate coefficient of determination.

^b^∆ *R*^2^: *R*^2^_with control variables_− *R*^2^_without control variables_.

^c^ƒ^2^: Cohen ƒ^2^.

**Table 8:** Path coefficients by bootstrapping.

Effect		Path coefficients (SD)	*P* value
**Direct effects**			
	PPCOM^a^→PQIHI^b^	0.703 (0.038)	.000
	PPCOM→DMP^c^	0.623 (0.038)	.000
	PPCOM→PPCON^d^	0.687 (0.035)	.000
	PQIHI → PC^e^	0.215 (0.066)	.001
	DMP→PC	0.094 (0.045)	.04
	PPCON→PC	0.209 (0.082)	.010
	PPCOM→PC	0.339 (0.067)	.000
**Total effects**			
	PPCOM→PC	0.693 (0.035)	.000

^a^PPCOM: Physician-patient communication.

^b^PQIHI: Perceived quality of internet health information.

^c^DMP: Decision-making preference.

^d^PPCON: Physician-patient concordance.

^e^PC: Patient compliance.

To further evaluate the mediating effects in the research model, we conducted an additional analysis using the bootstrapping method (n=5000, 95% CI). As shown in Table 8, the total effect of physician-patient communication on patient compliance was significant, and the direct effects of physician-patient communication on the 3 mediators and of the 3 mediators on patient compliance were all significant. Therefore, we used the Sobel test to assess the mediating role played by the 3 mediators between physician-patient communication and patient compliance. For the perceived quality of internet health information, the value of *Z*_perceived quality of internet health information_ was 3.028, which was significantly greater than 1.960, indicating that the mediation of perceived quality of internet health information was significant. In addition, for decision-making preference, the value of *Z*_d__ecision-making preference_ was 2.072, which was significantly greater than 1.960, indicating that the mediation of decision-making preference was significant. Furthermore, for physician-patient concordance, the value of *Z*_physician-patient concordance_ was 2.528, which was significantly greater than 1.960, indicating that the mediation of physician-patient concordance was significant. We can thus conclude that the perceived quality of internet health information, decision-making preference, and physician-patient concordance all played a partially mediating role between physician-patient communication and patient compliance, given that the direct effect of physician-patient communication on patient compliance was significant.

## Discussion

### Principal Findings

This study is the first that explores the impact of physician-patient communication in OHCs on patient compliance, and it makes theoretical contributions and practical implications for future studies on physician-patient communication and for guiding patient compliance through OHCs from the perspective of psychology. First, we constructed a research model to clarify the mechanisms through which physician-patient communication in OHCs impacts patient compliance by employing the self-determination theory. Previous studies have mainly focused on the relationship between offline physician-patient communication and physician-patient relationship and health outcomes, whereas the impact of communication in OHCs on patient compliance remains to be more focused, as OHCs are still in the stage of development, especially in China. Therefore, this study enriches theoretical researches on OHCs and patient compliance and improves the deficiencies of studies on strengthening patient compliance through communication in OHCs in China. In addition, this study used the self-determination theory to promote hypotheses and identify the motivation of patient compliance from the perspective of OHCs, and it enriches the application of the self-determination theory in the field of health-related behavior. The communication between physicians and patients in OHCs indirectly and positively affects patient compliance through the mediations of the perceived quality of internet health information, decision-making preference, and physician-patient concordance. Therefore, physician-patient communication in OHCs is beneficial for improving patient compliance.

Second, path coefficients from physician-patient communication to the 3 mediators are similar, and physician-patient communication just has a slightly stronger impact on the perceived quality of internet health information compared with decision-making preference and physician-patient concordance. Laugesen et al [30] validated that internet health information quality exerted a weak impact on physician-patient concordance. We speculated that physician-patient communication may have an indirect impact on physician-patient concordance through the mediation of perceived quality of internet health information; thus, the direct impact of physician-patient communication on physician-patient concordance was slightly weaker than that of physician-patient communication on the perceived quality of internet health information. The main purpose of patients using OHCs is to seek health-related information, including physicians, therapies, medicine, and other medical knowledge. As an important form of using OHCs, the communication of patients with physicians significantly affects patients’ perceived quality of internet health information. Moreover, we identified the significant impact of the perceived quality of internet health information on patient compliance, which was supported by Laugesen et al [30], who also claimed that high-quality internet health information can help enhance patient compliance. Furthermore, physicians are required to share high-quality information with their patients and guarantee patients’ perceived quality of information through communication in OHCs. OHCs must focus on the quality of their published health information. On the one hand, OHCs should strengthen the management of internet health information quality, which involves not only information itself but also users who publish and share information with other users. On the other hand, OHCs can conduct investigations to obtain feedback from their users so that they can understand the gap between the actual and perceived quality of information and then make efforts to improve the perceived information quality.

Third, the path coefficient from a patient’s decision-making preference to patient compliance is the smallest among relationships from the 3 mediators to patient compliance, and the path coefficients from the other 2 mediators to patient compliance are similar, implying that the impact of patient’s decision-making preference on patient compliance is weaker than the impact of the perceived quality of internet health information and physician-patient concordance on patient compliance. This finding is similar to the results of Laugesen et al [30], who confirmed that physician-patient concordance strongly, positively, and directly impacts patient compliance. Moreover, they did not determine the strong impact of internet health information quality on patient compliance, which is different from our finding. We speculated that this is because we discussed the direct impact of the perceived quality of internet health information, whereas Laugesen et al [30] considered the indirect impact. In addition, we narrowed the research background into OHCs. Findings suggest that physician-patient concordance is also a significant perspective, which promotes patient compliance by improving physician-patient communication in addition to patients’ perceived quality of internet health information. Physicians can seek concordance with patients in treatments to improve patient compliance. For instance, physicians can explain benefits, risks, and costs of treatment options [68] to patients, as a gap of medical knowledge may exist between physicians and patients; it may be useful to reduce the difference in cognition and increase concordance. Encouraging patients to tell their health conditions and express their attitudes toward treatments can help physicians learn additional information about patients and propose suitable recommendations, which may achieve an increased sense of identity from patients. In addition, physicians are encouraged to strengthen the emotional connection with patients to provide emotional autonomy support, which can satisfy patients’ psychological needs and improve patient compliance [75].

Finally, compared with the perceived quality of internet health information and physician-patient concordance, decision-making preference shows the weakest effect on the relationship between physician-patient communication and patient compliance, but it can also be a perspective to improve patient compliance. To maintain high compliance, physicians can encourage their patients to participate in decision making in OHCs. Although patients may be unable to assist in making any decision, physicians are required to inform patients of medical options and understand patients’ real ideas related to decision making. In that case, patients can perceive the support of autonomy in treatments and think that the decision that they follow is partly made by themselves and then be likely to comply with the treatment.

### Limitations

Several limitations and prospects in this study must be considered. First, this study used the perceived quality of internet health information, decision-making preference, and physician-patient concordance as mediators, and other variables can be discussed in future studies. Second, the development of health care is special in China because of its large population and uneven distribution of medical resources. The effect of OHCs on health care in China may be different from that in other countries. Therefore, the similarities and differences between China and other countries can be explored in further studies. Third, this study only collected data through a cross-sectional survey once; hence, we were unable to dynamically capture the changes of participants’ attitudes toward all variables. Finally, we only matched the sample with the characteristics of typical OHCs’ users but did not consider the Chinese population. We originally intended to consider Chinese census data but found it difficult because of China’s large population. Ultimately, we believe that future studies may be able to address this issue.

### Conclusions

This study indicates that physician-patient communication in OHCs positively impacts patient compliance through mediations of the perceived quality of internet health information, decision-making preference, and physician-patient concordance. In our research model, physician-patient communication shows similar effects on the perceived quality of internet health information, decision-making preference, and physician-patient concordance, and patient’s decision making has the weakest impact on patient compliance compared with the other 2 mediators. In terms of the mediation, all 3 mediators play a partially mediating impact on the relationship between physician-patient communication and patient compliance. In addition, these findings suggest the following: (1) physicians can share high-quality health information with patients, ask patients’ real opinions about information, and make efforts to decrease patients’ misunderstanding of information; (2) physicians can discuss benefits, risks, and costs of treatment options with patients, encourage patients to express their attitudes and participate in health-related decision making, and strengthen the emotional connection with patients to provide emotional autonomy support in OHCs; and (3) OHCs can not only strengthen the management of their published health information quality but also understand users’ actual attitudes toward information quality and then try to reduce the gap between the perceived and actual quality of information.

## Appendix

Multimedia Appendix 1 Measurement instruments.

## Abbreviations

AVE: average variance extracted
CR: composite reliability
OHC: online health community
PLS: partial least squares
SEM: structural equation modeling
