# Supplementary material for: Impact of Physician-Patient Communication in Online Health Communities on Patient Compliance: Cross-Sectional Questionnaire Study
Source: J Med Internet Res. 2019 May 13;21(5):e12891. doi: 10.2196/12891 (PMC6535977; doi:10.2196/12891)
Supplement: Multimedia Appendix 1 [file jmir_v21i5e12891_app1.doc]

## Multimedia Appendix 1

Table A1. Measurement Instruments.

| **Construct** | **Scale/Scoring** | **Items** |
| --- | --- | --- |
| Physician-patient Communication [77] | - 5-point Likert - Strongly disagree to strongly agree | 1. In online health communities, physicians greeted me in a way that made me feel comfortable. |
|  |  | 2. In online health communities, physicians treated me with respect. |
|  |  | 3. In online health communities, physicians showed interest in my ideas about my health. |
|  |  | 4. In online health communities, physicians understood my main health concerns. |
|  |  | 5. In online health communities, physicians paid attention to me. |
|  |  | 6. In online health communities, physicians let me talk without interruptions. |
|  |  | 7. In online health communities, physicians gave me as much information as I wanted. |
|  |  | 8. In online health communities, physicians talked in terms I could understand. |
|  |  | 9. In online health communities, physicians checked to be sure I understood everything. |
|  |  | 10. In online health communities, physicians encouraged me to ask questions. |
|  |  | 11. In online health communities, physicians involved me in decisions as much as I wanted. |
|  |  | 12. In online health communities, physicians discussed next steps. |
|  |  | 13. In online health communities, physicians showed care and concern. |
|  |  | 14. In online health communities, physicians spent the right amount of time with me. |
| Perceived Quality of Internet Health Information [21] | - 5-point Likert - Strongly disagree to strongly agree | **Relevance:** |
|  |  | 1. For your health information needs, to what degree do you believe the Internet Health Information provided by the website was applicable to your needs? |
|  |  | 2. For your health information needs, to what degree do you believe Internet Health Information provided by the website was related to your needs? |
|  |  | 3. For your health information needs, to what degree do you believe Internet Health Information provided by the website was pertinent to your needs? |
|  |  | 4. For your health information needs, to what degree do you believe Internet Health Information provided by the website was relevant to your needs? |
|  |  | **Understandability:** |
|  |  | 1. For your health information needs, to what degree do you believe Internet Health Information provided by the website was clear in meaning? |
|  |  | 2. For your health information needs, to what degree do you believe Internet Health Information provided by the website was easy to read? |
|  |  | 3. For your health information needs, to what degree do you believe Internet Health Information provided by the website was easy to comprehend? |
|  |  | 4. For your health information needs, to what degree do you believe Internet Health Information provided by the website was understandable? |
|  |  | **Adequacy:** |
|  |  | 1. For your health information needs, to what degree do you believe Internet Health Information provided by the website was sufficient? |
|  |  | 2. For your health information needs, to what degree do you believe Internet Health Information provided by the website was complete? |
|  |  | 3. For your health information needs, to what degree do you believe Internet Health Information provided by the website was adequate? |
|  |  | 4. For your health information needs, to what degree do you believe Internet Health Information provided by the website contained the necessary topics/categories? |
|  |  | **Usefulness:** |
|  |  | 1. For your health information needs, to what degree do you believe Internet Health Information provided by the website was informative? |
|  |  | 2. For your health information needs, to what degree do you believe Internet Health Information provided by the website was valuable? |
|  |  | 3. For your health information needs, to what degree do you believe Internet Health Information provided by the website was helpful? |
|  |  | 4. For your health information needs, to what degree do you believe Internet Health Information provided by the website was useful? |
| Decision-making Preference [78] | - 5-point Likert - Strongly disagree to strongly agree | 1. The important medical decisions should be made by the doctor, not by you. |
|  |  | 2. You should go along with your doctor’s advice even if you disagree with it. |
|  |  | 3. When hospitalized, you should not be making decisions about your own care. |
|  |  | 4. You should feel free to make decisions about everyday medical problems. |
|  |  | 5. If you were sick, as your illness became worse you would want the doctor to take greater control. |
|  |  | 6. You should decide how frequently you need a checkup. |
| Physician-patient Concordance [21] | - 5-point Likert - Strongly disagree to strongly agree | 1. To what extent do you think the doctor understood why you came in to see him/her? |
|  |  | 2. To what extent did you and the doctor agree on the diagnosis with respect to the significant health situation? |
|  |  | 3. To what extent did you and the doctor agree on what part you play in making decisions about the significant health situation? |
|  |  | 4. To what extent did you and the doctor agree on the recommended treatment for the significant health situation? |
|  |  | 5. To what extent did you and the doctor agree on the possible outcomes associated with the recommended treatment for the significant health situation? |
| Patient Compliance [21] | - 5-point Likert - Strongly disagree to strongly agree | 1. I am following/did follow the doctor’s suggestions exactly. |
|  |  | 2. I am following/did follow the doctor’s drug/medication recommendations. |
|  |  | 3. I am following/did follow the doctor's orders, such as to stay in bed. |
|  |  | 4. I have returned or plan to return to the doctor on the schedule he/she suggested. |
|  |  | 5. I have had or plan to have the follow-up tests recommended by the doctor |
